# HAIR CORTISOL FEASIBILITY AND DEMOGRAPHIC CORRELATES IN A SAMPLE OF OLDER ADULTS FROM PUERTO RICO

**DOI:** 10.1093/geroni/igac059.2412

**Published:** 2022-12-20

**Authors:** Erin Ballard, Bulent Turan, Michael Crowe

**Affiliations:** University of Alabama at Birmingham, Hoover, Alabama, United States; Koc University, Sariyer, Istanbul, Turkey; University of Alabama-Birmingham, Birmingham, Alabama, United States

## Abstract

Hair cortisol is increasingly being used as a biomarker of chronic HPA-axis activation. Studies using older adults often exclude a substantial portion of participants due to insufficient hair or non-detectable cortisol levels, but do not provide details on correlates of these factors. We examined feasibility of hair measurement and cortisol detectability in an ongoing study of older adults in Puerto Rico. Among the first 537 participants in the current follow-up of the Puerto Rican Elder Health Conditions (PREHCO) study (now age 78-106 years old), approximately 11% of participants refused to give a hair sample and 20% did not have enough hair to sample. Women (13%) were significantly more likely than men (4%) to refuse hair collection. However, men (47.7%) were significantly more likely than women (4.8%) to not have enough hair. Of participants with enough hair to take a sample (n=372), 23% had non-detectable levels of cortisol. Black participants were the most likely to have non-detectable hair cortisol (43%), followed by multiracial mestizo (32%). The two most common racial categories in our sample, multiracial trigueño (23%), and white (17%) were the least likely to have non-detectable cortisol. In terms of hair products (including frequency of hair washing and use of conditioner, dye, or perm), only the use of chemical hair straighteners was associated with higher likelihood of non-detectable cortisol (38%). Findings underscore the importance of measuring hair products when examining hair cortisol in older adults and suggest that older black participants may be disproportionately excluded due to non-detectable cortisol levels.

